# Overweight and Body Image Perception in Adolescents with Triage of Eating Disorders

**DOI:** 10.1155/2017/8257329

**Published:** 2017-08-10

**Authors:** Roberta Stofeles Cecon, Sylvia do Carmo Castro Franceschini, Maria do Carmo Gouveia Peluzio, Helen Hermana Miranda Hermsdorff, Silvia Eloiza Priore

**Affiliations:** Universidade Federal de Viçosa, Viçosa, MG, Brazil

## Abstract

**Purpose:**

To verify the influence of overweight and alteration in the perception of the corporal image during the triage of eating disorders.

**Method:**

A food disorder triage was performed in adolescents with 10 to 19 years of age using the Eating Attitudes Test (EAT-26), Children's Eating Attitudes Test (ChEAT), and Bulimic Investigatory Test Edinburgh (BITE), as well as a nutritional status evaluation. The perception of body image was evaluated in a subsample of adolescents with 10 to 14 years of age, using the Brazilian Silhouette Scale. The project was approved by the Human Research Ethics Committee of the Federal University of Viçosa, Minas Gerais, Brazil.

**Results:**

The prevalence of eating disorder triage was 11.4% (*n* = 242) for the 2,123 adolescents evaluated. Overweight was present in 21.1% (*n* = 447) of the students, being more prevalent in the early adolescence phase, which presented levels of distortion of 56.9% (*n* = 740) and dissatisfaction of 79.3% (*n* = 1031). Body dissatisfaction was considered as a risk factor, increasing by more than 13 times the chance of TA screening.

**Conclusion:**

Overweight was correlated with the ED triage and body dissatisfaction was considered as a risk factor, increasing the chances of these disorders by more than 13 times.

## 1. Introduction

Adolescence consists of the period from 10 to 19 years of age, according to World Health Organization (WHO), and is marked by intense growth and development and physical, psychological, social, and emotional changes that result in behavioral modifications by the adolescents [1].

At present, the eating and behavioral patterns of adolescents are characterized by high-energy (mainly fat) food, omission of daily meals, low consumption of fruits and vegetables, and an increasingly sedentary routine with many hours in front of the computer and television screen and fewer hours of physical exercise. This results in a greater effort to maintain a lean body and the ideal standard of imposed beauty spread by the media and society [1].

Overweight is already a public health problem and affects one-fifth of adolescents in Brazil and more than nine million children and adolescents in the United States. Obesity during adolescence is associated with morbidities during this phase and also throughout adult life, with eating disorders (ED) being the third most common chronic disease in adolescence, losing only to obesity and asthma [2, 3].

The ED are pathological conditions of multidimensional etiology which determine a distorted relationship between the individual, their eating behavior, and body shape [4]. Self-esteem and body dissatisfaction are considered predictors of ED and adolescence is a crucial phase for the positive or negative development of body image [5].

Distortion and dissatisfaction with body image may form a link between overweight and obesity, as excessive preoccupation with appearance and unremitting pursuit of the perfect, lean body can generate negative feelings and devaluation, resulting in change in the eating behavior, leading to overweight or the development of ED [6].

Therefore, the goal of the study was to verify in the triage of ED the influence of the overweight and the alteration in the perception of the body image.

## 2. Methodology

The research performed was a cross-sectional study conducted with 2,123 adolescents between 10 and 19 years of age, of both sexes, from public and private schools in the city of Viçosa, Minas Gerais, Brazil.

After contact with the Education Department of the municipality, all the schools offering primary and secondary education were sought out to clarify the project's goals and request authorization for it. With only 1 refusal, we were able to evaluate 23 schools.

Data collection took place in a school setting, in a reserved place, after the delivery of the Informed Consent Form (ICF), signed by the adolescent and his/her parents/guardians. Those below 18 years of age also delivered the Term of Consent with the teenager's own signature.

The questionnaire was applied with the inclusion criteria (ages between 10 and 19 years; males having to answer as to the presence of axillary hairs; presence of the menstrual cycle, contraceptive use for less than 2 months, and possible pregnancy for females; and not having a pacemaker or prosthesis), anthropometric profile evaluation, and the application of the ED triage questionnaires.

The anthropometric profile was evaluated by weight and height according to the Jellife (1968) [7] criteria and the calculation of Body Mass Index (BMI), and the nutritional status was assessed according to World Health Organization (WHO) (2007) [8].

The ED triage was performed with three questionnaires: the Eating Attitudes Test (EAT-26), answered by adolescents with ages from 13 to 14, with a cut-off point equal to or greater than 20 points for positive screening; Children's Eating Attitudes Test (ChEAT), an EAT-26 adaptation, with easy-to-understand language, answered by adolescents with 10 to 12 years of age, using the same cut-off point; and the Bulimic Investigatory Test Edinburgh (BITE), answered by all adolescents, with scores greater than or equal to 15 for ED triage [9–11].

The body image perception was evaluated in a subsample of adolescents with 10 to 14 years of age, using the Brazilian Silhouette Scale. For adolescents older than 13 years, the scale was composed of 15 cards with mean BMI values of 12.5 to 47.5 kg/m^2^, with an interval of 2.5 kg/m^2^ for each card. For adolescents younger than 13 years, the scale was used with 11 cards presenting mean BMI values of 12 to 29 kg/m^2^ and an interval of 1.7 kg/m^2^ for each card [12].

To calculate body image distortion and dissatisfaction, the Brazilian Silhouette Scale has two questions regarding the cards: “Which figure best represents your body today?” regarding the current BMI and “Which figure shows the body you would like to have?” which evaluates the desired BMI [13].

The distortion was calculated by the difference between the current BMI value and the actual BMI (weight to height ratio squared). Adolescents with null values of this difference were considered undistorted, and those with positive values (overestimation of the body) and negative values (underestimation of the body) were distorted [12, 13].

Body dissatisfaction was calculated by the difference between the desired BMI and the current BMI. The adolescents who presented null values for this difference were satisfied with their body and dissatisfied with the objective of increasing their body size. As for those who presented positive values, they were unsatisfied and wanted to decrease body size [12, 13].

We performed statistical analyses using Statistical Package for Social Sciences (SPSS) version 21.0 and Data Analysis and Statistical Software (STATA) version 11.0.

Descriptive statistics, correlation, chi-square, and logistic regression were performed. The project was approved by the Committee of Ethics in Research with Human Beings of the Federal University of Viçosa, with the opinion number 1,443,922.

## 3. Results

The ED triage and anthropometric evaluation were performed in 2,123 adolescents aged between 10 and 19 years, which represented 19.9% of the total number enrolled from the urban area of the municipality. Of the total, 66.5% (*n* = 1,411) were female and their median age was 13.48 ± 2.48 years. Positive triage for ED, considering an increased score for at least one of the questionnaires used, was 11.4% (*n* = 242).

The prevalence of positive screening for ED was higher in the final phase of adolescence (17 to 19 years), with values of 20.2% (*n* = 60), and then when compared to the intermediate phase (14 to 16 years), with 18.0% (*n* = 128), and to the initial phase (10 to 13 years), with prevalence of 4.9% (*n* = 54). Evaluating the female and male adolescents, the first group presented prevalence of 15.0% (*n* = 211) of positive screening for ED, in contrast with 4.4% (*n* = 31) of the second group.

In relation to nutritional status, we identified 21.1% (*n* = 447) of overweight cases, according to the Body Mass Index by Age (BMI/A), prevalence considered “high exposure” by World Health Organization (WHO). The prevalence of overweight was higher in the early stage of adolescence, with 26.7% (*n* = 297), and decreased as adolescence advanced, with 15.2% (*n* = 109) and 13.8% (*n* = 41) in the intermediate and final phases, respectively.

When the nutritional status was assessed between the groups with and without positive triage for ED, the first one showed prevalence of overweight of 38.0% (*n* = 92) versus 18.9% (*n* = 355) in the second group.

The presence of secondary sexual characteristics (presence of axillary hair in boys and the menstrual cycle in girls) was 65.0% (*n* = 1,379) in adolescents: 60.8% (*n* = 433) and 67.0% (*n* = 946) in males and females, respectively.

After performing the triage, the EAT-26, ChEAT, and BITE questionnaires, used to evaluate signs and symptoms of anorexia and bulimia nervosa, were assessed for the highest scores. For EAT-26 and ChEAT, the question with the highest score (“Always” = 3 points) was number 14, related to the concern of having fat in the student's body, with a frequency of 21.6% (*n* = 459). Therefore, it showed that more than one-fifth of these adolescents are concerned with body image.

These questionnaires can also be divided into three scales: the Diet Scale, which reflects the often pathological refusal to eat high-calorie foods and an intense preoccupation with body shape; the Bulimia Scale and Food Concern, which is related to the presence of episodes of binge eating, followed by compensatory methods to avoid weight gain; and, lastly, the Oral Control Scale, referring to self-control in relation to foods and social factors that encourage their ingestion.

The Diet Scale had a median of 6 points, a minimum of 0 points, and a maximum of 36 points, followed by the Oral Control Scale, with a median of 3 points, a minimum of 0 points, and a maximum of 18 points, and the Bulimia Scale and Concern with Food, with a median of 1 point, a minimum of 0 points, and a maximum of 18 points. When analyzed in each adolescence phase, the initial phase scored higher (6 points) in relation to the Dietary Scale when compared to the intermediate phase (*p* < 0.001) and late adolescence (*p* < 0.001), both with 4 points.

The higher score on the Diet Scale reveals the adolescents' preoccupation with food and its caloric content and once again the concern with body shape, which presents itself increasingly in younger groups of adolescents.

In the BITE questionnaire, the questions with the highest score (1 point) were numbers 1 and 16, which ask the adolescents about their daily dietary pattern and if the thought of becoming fat terrifies them, with frequencies of 52.7% (*n* = 1,118) and 52.8% (*n* = 1,121), respectively. Once again, we can see the growing concern of these adolescents with issues related to food and body image.

The perception of the body image was evaluated in a subsample of adolescents with 10 to 14 years of age. Body distortion was found in 56.9% (*n* = 740) of adolescents, and 416 of them overestimated body size. Body dissatisfaction was present in 79.3% (*n* = 1,031) of them, with 677 students showing a desire to decrease body size.

Evaluating the body image of this subsample, in the groups with and without screening for ED, there was a distortion of 71.8% (*n* = 51) in the first group against 56.1% (*n* = 689) in the second and body dissatisfaction of 90: 1% (*n* = 64) in adolescents with positive screening against 71.8% (*n* = 882) in the others. What is impressive is that more than half of the students who presented body distortion and dissatisfaction overestimated body shape and wished to decrease body size.

There was a correlation between anthropometric profile and body image perception with the scores from the questionnaires used in the ED triage. The weight was correlated with all questionnaires, presenting values of *r* = 0.058 (*p* < 0.001) for EAT-26 and ChEAT and *r* = 0.217 (*p* < 0.001) for BITE, as well as BMI, with values of *r* = 0.187 (*p* < 0.001) for EAT-26 and ChEAT and *r* = 0.299 (*p* < 0.001) for BITE. This showed that the overweight correlated positively with the score of the ED triage questionnaires.

Body image also correlated positively with the ED triage questionnaires, showing that adolescents who displayed body image distortion, overestimating their body shape, presented higher BITE scores (*r* = 0.159; *p* < 0.001) and those who were dissatisfied, wanting to decrease their body size, obtained higher scores in EAT-26 and ChEAT (*r* = −0.0272; *p* < 0.001) and in BITE (*r* = −0.0239; *p* < 0.001).

Secondary sexual characteristics, an attribute of puberty, were associated with positive ED triage (*χ*^2^ = 68.47; *p* < 0.001), as well as nutritional status, according to BMI/A (*χ*^2^ = 4.90; *p* < 0.001), which indicates that the own body changes during the adolescence and the increase of the weight can be risk factors for the development of ED. Body image was also associated with positive triage for ED, with values of *χ*^2^ = 3.39 (*p* = 0.001) for distortion and *χ*^2^ = 5.80 (*p* < 0.001) for dissatisfaction.

The anthropometric and body image perception variables were used in the logistic regression model. Weight and BMI were considered as risk factors for the positive screening for ED, increasing by 1.05 and 1.23 times the chance of this triage. Regarding body image distortion, overestimation of body shape was a risk factor, increasing by 2.55 times the chance of signs and symptoms related to ED, and body image dissatisfaction related to the desire to decrease body size increased by 13.45 times the chance of this disorder. Overweight and body image dissatisfaction resulting from this alteration in the nutritional status may serve as triggering factors and even maintainers of ED.

## 4. Discussion

The prevalence of positive triage for ED of 11.4% (*n* = 242) is close to those found in the literature. Researches conducted in different countries, with different ED triage questionnaires, each with a specific cut-off point, found signs and symptoms of anorexia and bulimia in adolescents ranging from 17.3% to 37% [14–18].

Alvarenga et al. (2010) evaluated adolescents and young adults with 18 to 50 years of age in the five different regions of Brazil (North, Northeast, Center West, Southeast, and South), all from public and private educational institutions, finding 26.1% of them with risk behavior for ED. The prevalence ranged from 23.7% to 30.1% among the five regions of the country and the questionnaire used to assess the risk behavior for ED was the EAT-26, considering as a cut-off point the value greater than or equal to 21 points [14, 15].

Alves et al. (2012) applied the EAT-26 and BITE to 365 children and adolescents aged 7 to 14 years. The cut-off points used for positive screening of ED were different from the present study, with scores greater than 21 for EAT-26 and greater than or equal to 20 for BITE, identifying presence of ED symptoms in 23.0% of the students [16].

Castrillón et al. (2012) evaluated adolescents in 8th grade to 11th grade of private schools by using the Eating Disorders Inventory 2 (EDI-2) questionnaire with a cut-off point greater than 14 points. The prevalence of positive screening for ED was 24.7%, a higher value compared to a study carried out with university students in the same city in 2007, with prevalence of 17.3% [17]. Disotuar et al. (2015) also reported a higher incidence of risky eating behavior in adolescents aged 15 to 19 years, with prevalence of up to 37%. The authors used the criteria of the Diagnostic and Statistical Manual of Mental Disorders (DSM-V) in adolescents below 16 years of age, which may lead to a more accurate diagnosis of these disorders [18].

The ED have a higher incidence in the intermediate and final phases of adolescence (15 to 19 years), but it is already possible to find signs and symptoms of these eating disorders in the school and early adolescence phases, as shown in the present study [19]. The prevalence of positive screening for ED was higher in female adolescents, a result that is in agreement with data from the literature, which shows that the frequency of risky food behavior to ED is higher in adolescents and young women [18].

The high exposure of overweight was similar to the results of the Pesquisa de Orçamentos Familiares 2008-2009 (POF) and the Pesquisa Nacional de Saúde do Escolar (PeNSE) of 2009, carried out in Brazil. In POF, the prevalence of overweight among adolescents with 10 to 19 years of age was 20.5% and, in PeNSE, adolescents with 13 to 15 years of age presented 23.2% of this nutritional problem [20, 21].

The overweight distribution according to the phases of adolescence was also similar to the HBS data, showing higher prevalence among the younger adolescents, reaching almost 30% of this nutritional change [20].

Overweight, especially obesity, has been considered a public health problem in the world epidemiological scenario, and since the 1970s it has increased considerably in Brazil, at increasingly young ages [22]. The physiological changes that occur in the puberty phase, with the increase of body fat, especially in the female sex, together with the changes in personality and altered nutritional status in one-fifth of the adolescent population, end up altering the perception of the body image and embedding the thought of a thin body in these adolescents [1].

Conti et al. (2005) evaluated the association of body dissatisfaction with a body satisfaction scale in adolescents with overweight from 10 to 14 years of age with regard to the areas of the stomach and waist (regions of fat accumulation) and body weight for the boys and in more regions as for the girls, such as the hair area, buttocks, hips, thighs, legs, stomach, and shoulders/back, muscle tone, body weight, and general aspects. This exposed the greater concern from girls with body shape and their greater suffering when they face weight increase and changes in the body shape [23].

A study carried out on Mexican children and adolescents with 8 to 11 years of age shows that overweight is related to body image dissatisfaction and low self-esteem, and 94% of them presented body image distortion and 100% presented dissatisfaction, wishing to decrease body size. More than half of them chose figures that characterized low weight [24].

Liberali et al. (2013) also found an association between altered body image perception and ED risk, showing that adolescents with distortion were 15 times more likely to present symptoms of these disorders [25]. In the present study, body dissatisfaction was also considered as a risk factor for ED screening, increasing by more than 13 times the chance of these signs and symptoms.

Body dissatisfaction and low self-esteem are considered to be predictors of ED, and body image perception has been a subject of interest in studies since the 1980s due to its relation to public health problems such as obesity, sedentary lifestyle, and ED [5, 26].

After assessing the association between weight, BMI, and body dissatisfaction and the score of the ED screening questionnaires and verifying that overweight contributes to the alteration of the body image perception, it becomes important to evaluate and understand this variable better once that body image is a multidimensional construction, encompassing both the physical structure of the body and the mental representation, perception, thoughts, and actions related to it [5].

Other characteristics associated with adolescent body dissatisfaction are those related to the puberty phase, such as menarche and the presence of hairs on the body. In the present study, the secondary sexual characteristics of puberty were associated with the ED screening, as in the study done by Scherer et al. (2010) who also found an association between the presence of menarche, body dissatisfaction, and symptoms of ED. Kaczmarek and Trambacz-Oleszak (2015) found an association between body dissatisfaction and different phases of the menstrual cycle in adolescents aged 12 to 18 years, showing that puberty and the body modifications it brings often result in bodily dissatisfaction, which can serve as a risk factor for ED [27, 28].

Therefore, the approach to adolescence-related issues, such as puberty, body changes (weight, height, and percentage of fat), psychological factors, and the way one perceives the body (body image), should be done with caution by professionals with the use of motivational interview techniques, avoiding languages that seem offensive or that may generate negative thoughts about the body of the adolescent, considering that this can be a risk factor for the development and maintenance of ED [3].

The questionnaires used to screen for ED revealed a higher frequency of responses related to the preoccupation with body image, and the EAT-26 and ChEAT questionnaires showed a greater median of points in the Diet Scale, which reflects the concern with body shape and with the caloric content of foods and the refusal, sometimes pathological, to ingest them.

The adolescent eating pattern is linked to the multiple changes that occur during this stage (biological, psychological, and sociocultural), characterized by excessive consumption of fast food, foods with high sugar content, salt, and fat, low intake of fruits and vegetables, and omission and individualization of meals and taking them in inappropriate places [25, 29, 30].

The media today plays an important role in the eating pattern of adolescents, since it stimulates a lean body pattern with no curves and delivers out antiobesity messages. However, at the same time, it promotes easy-to-prepare foods (high in fat and salt), fast food, and very sugary foods but with the false idea that these foods can be consumed without moderation and do not alter nutritional status if consumed in excess [30].

The adolescent eating pattern, coupled with the corporal modifications characteristic of this phase, can result in unhealthy behaviors, such as fad diets, omission of meals, ingestion of appetite moderators, prolonged fasting, and use of diuretics and laxatives in an attempt to “be healthier,” which may result in the development of an ED [3].

## 5. Conclusion

Overweight was correlated with the ED triage and body dissatisfaction was considered as a risk factor, increasing the chances of these disorders by more than 13 times. The prevention or even the treatment for overweight should be done in such a way that weight or body shape is not the main theme of this approach.

The health professional should involve the whole family of the adolescents in this process, maintaining the focus on their orientation in the change of behavior and lifestyle and offering conditions so that the adolescents can improve their nutritional state and their self-esteem with respect to their corporal image through a healthy approach, without encouraging any behavior that results in ED.
